# Supplementary figures and images for: From individuals to ancestries: Towards attributing trait variation to haplotypes
Source: PLoS Genet. 2025 Sep 30;21(9):e1011883. doi: 10.1371/journal.pgen.1011883 (PMC12507255; doi:10.1371/journal.pgen.1011883)

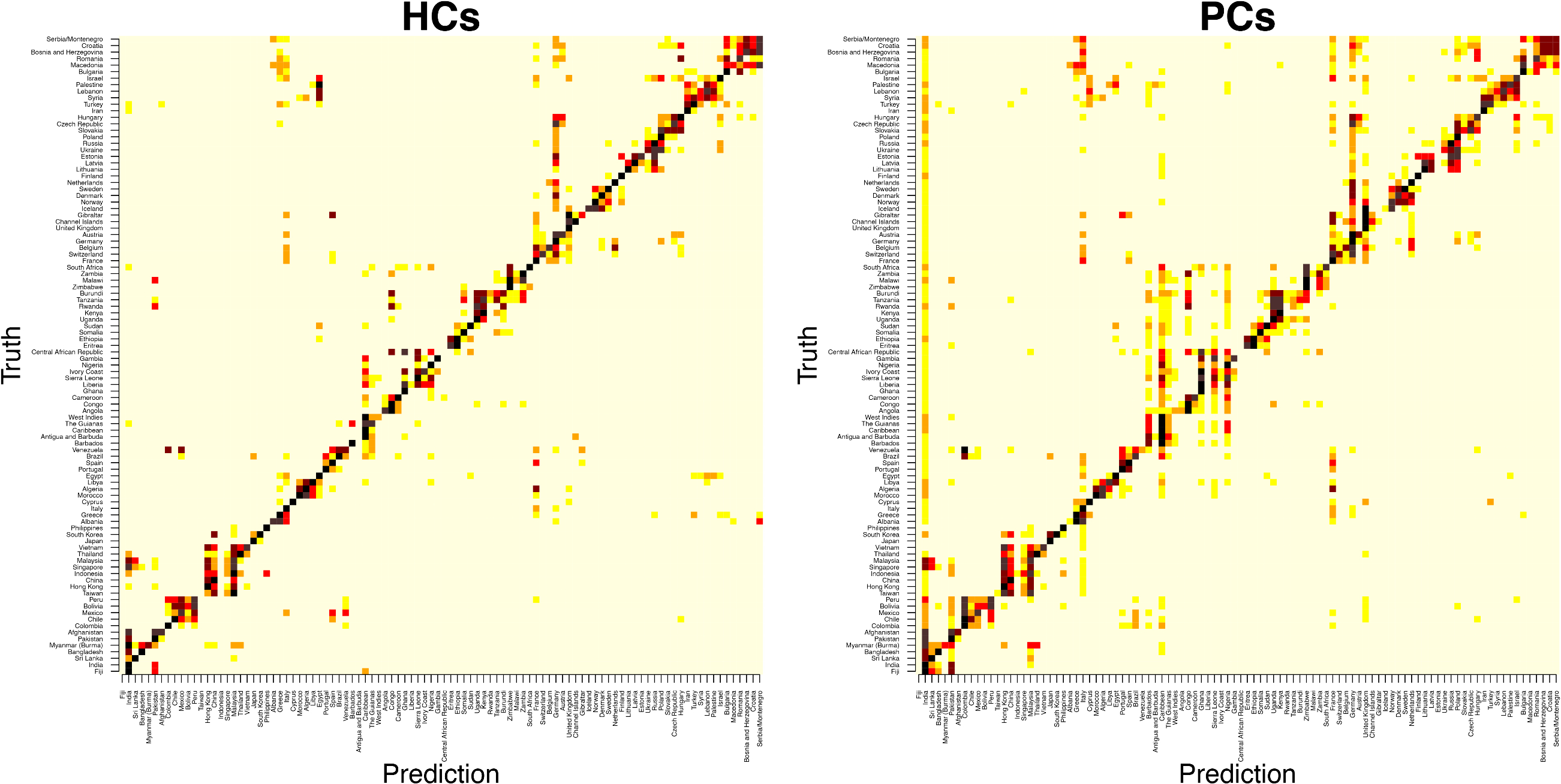

Supplement: S1 Fig — (TIFF) [file pgen.1011883.s005.tif]

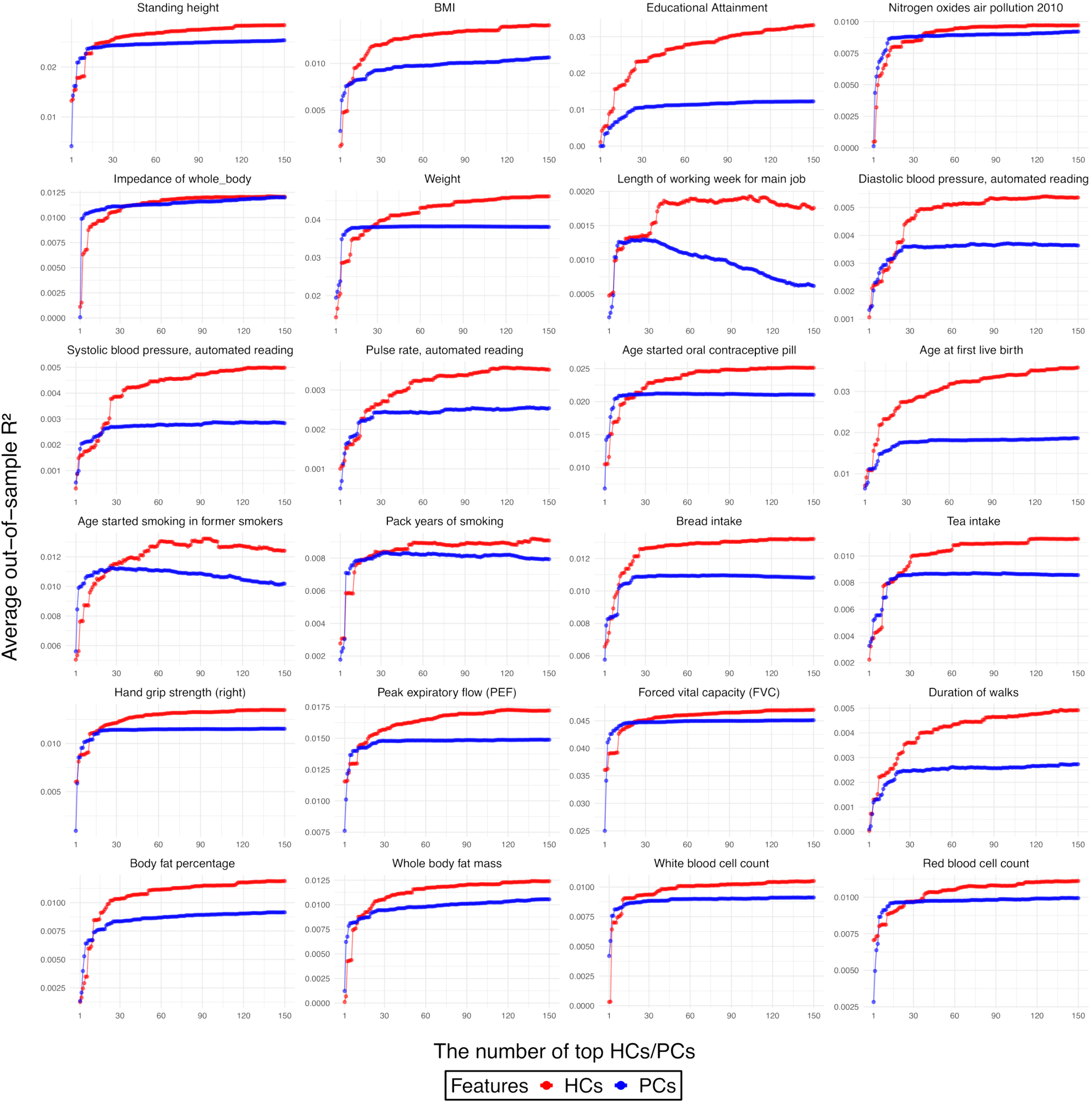

Supplement: S2 Fig — This study includes n = 406,773 UK Biobank individuals. (TIFF) [file pgen.1011883.s006.tif]

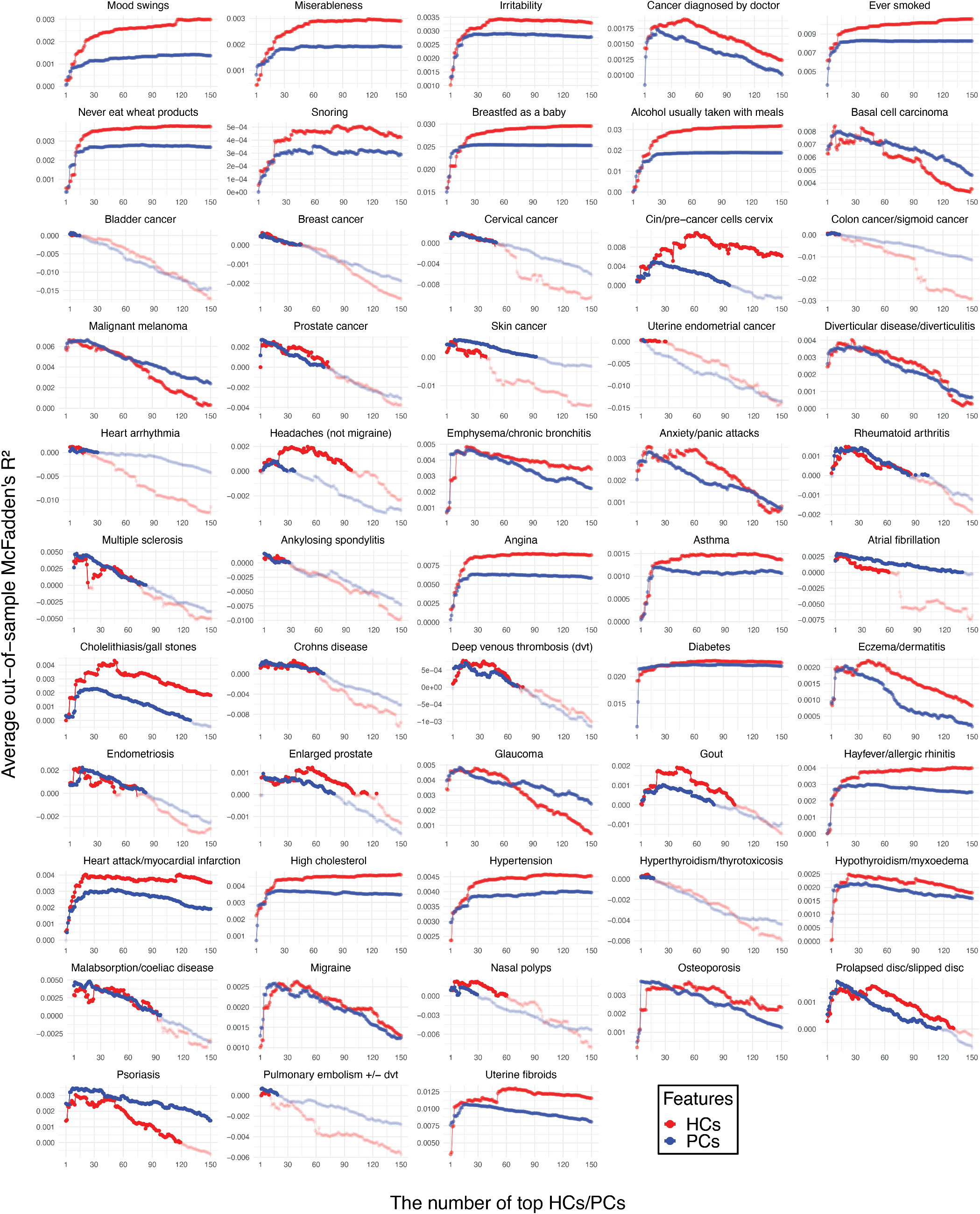

Supplement: S3 Fig — The points and lines are faded for negative average out-of-sample R2. This study includes n = 406,773 UK Biobank individuals. (TIFF) [file pgen.1011883.s007.tif]

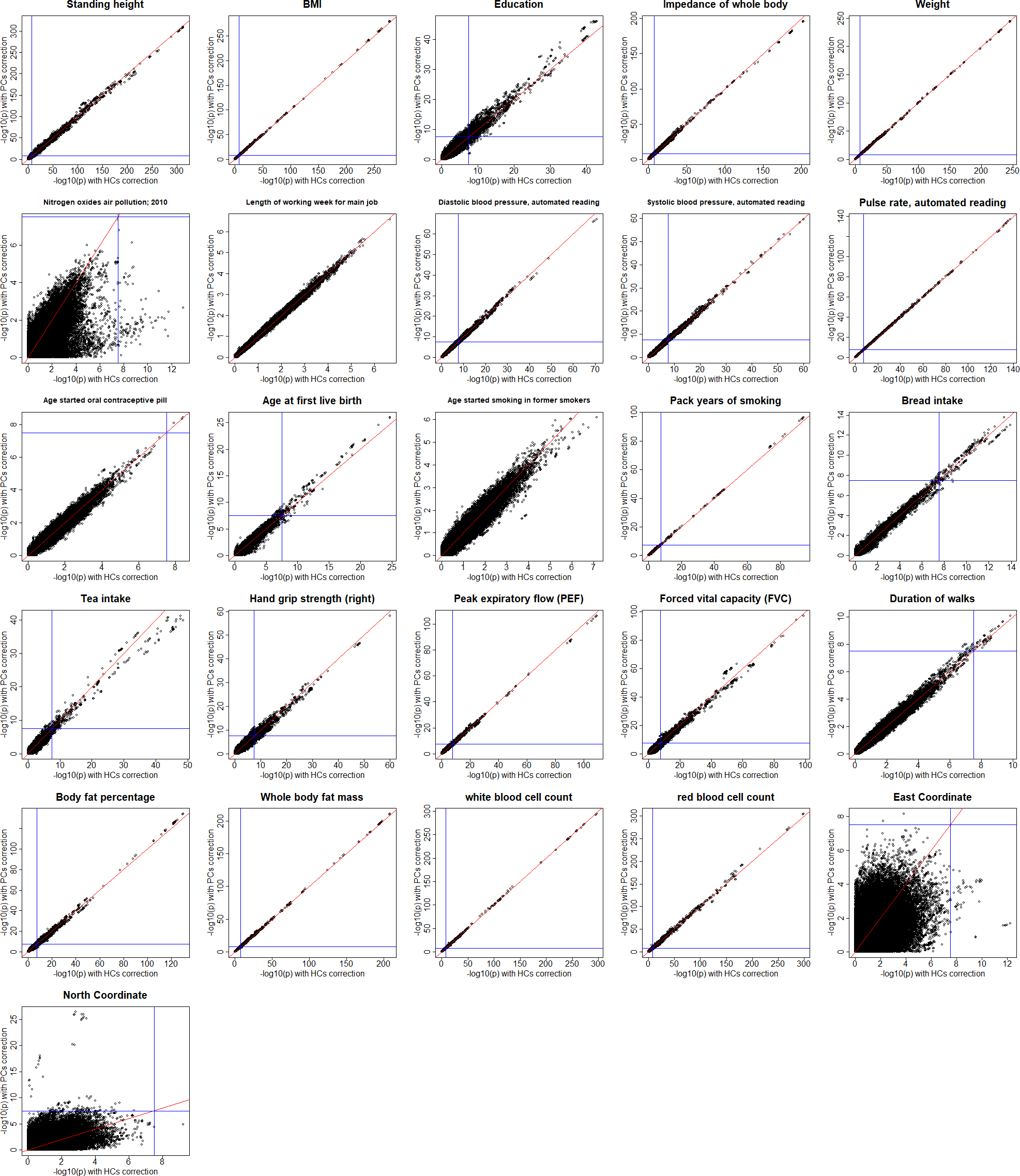

Supplement: S4 Fig — Each point represents its -log10(P-value) from GWAS corrected by the top 18 HCs or PCs. This study includes n = 406,773 UK Biobank individuals. (TIFF) [file pgen.1011883.s008.tif]

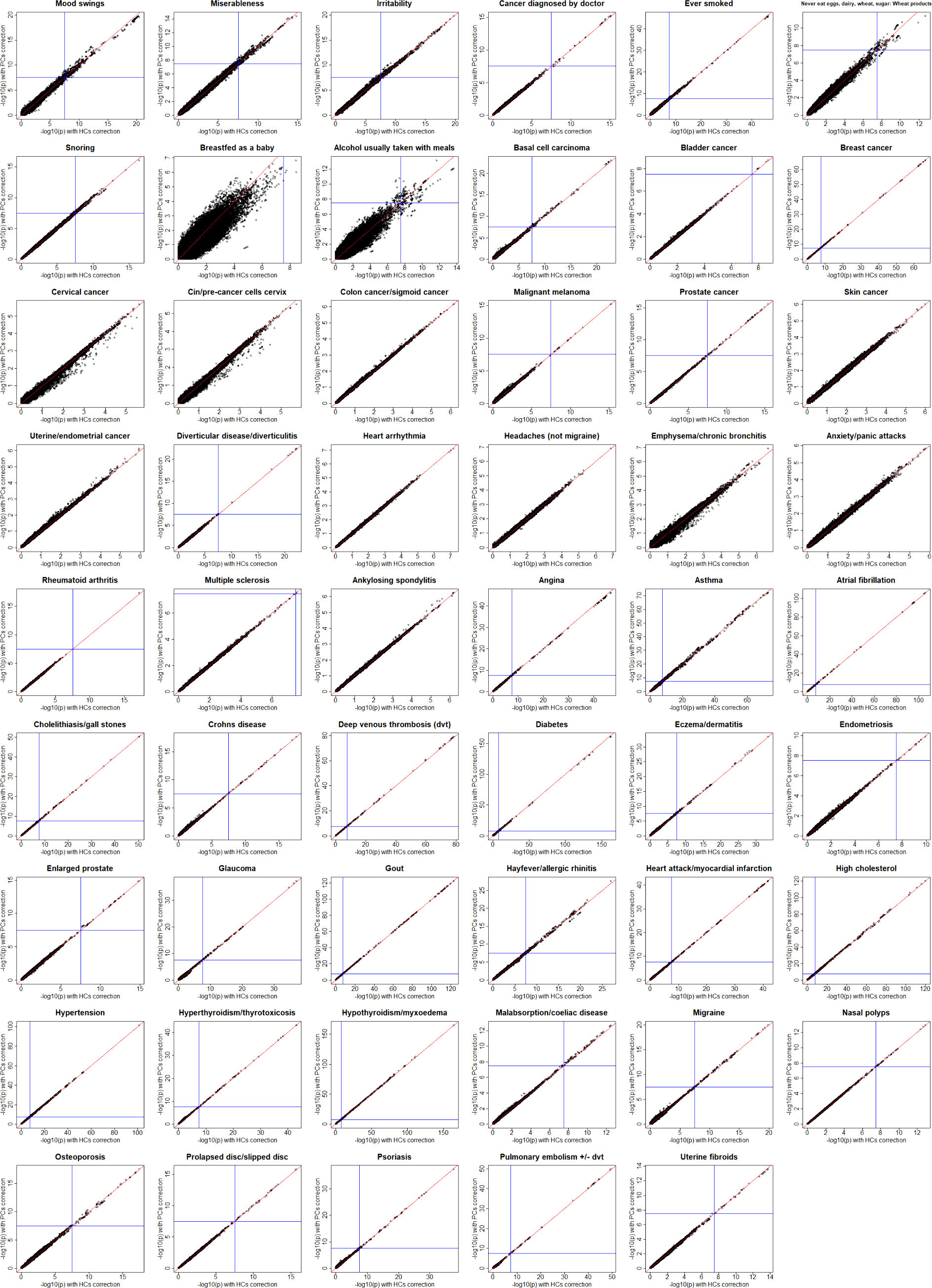

Supplement: S5 Fig — Each point represents its -log10(P-value) from GWAS corrected by the top 18 HCs or PCs. This study includes n = 406,773 UK Biobank individuals. (TIFF) [file pgen.1011883.s009.tif]

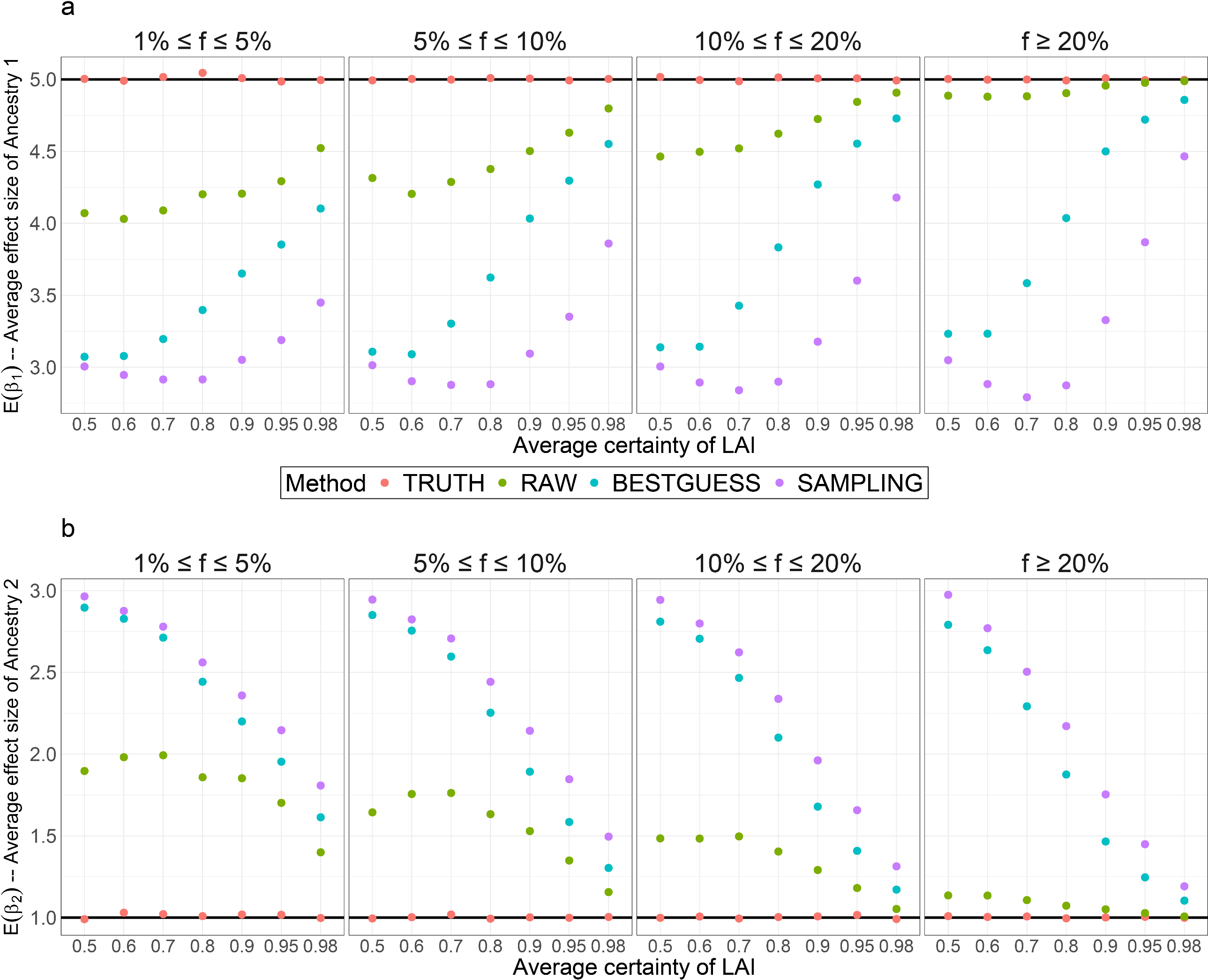

Supplement: S6 Fig — E(Ai1d*) denotes the average probability of Ancestry 1. The x-axis represents the average certainty of LAI (see Methods) and the y-axis represents the average estimated effect sizes for Ancestry 1 (plot a) and Ancestry 2 (plot b). Different MAF thresholds f are compared: 1%≤f≤5%, 5%≤f≤10%, 10%≤f≤20%, and f≥20%. The simulation was repeated 1,000 times with n = 20,000 diploid individuals. (TIFF) [file pgen.1011883.s010.tif]

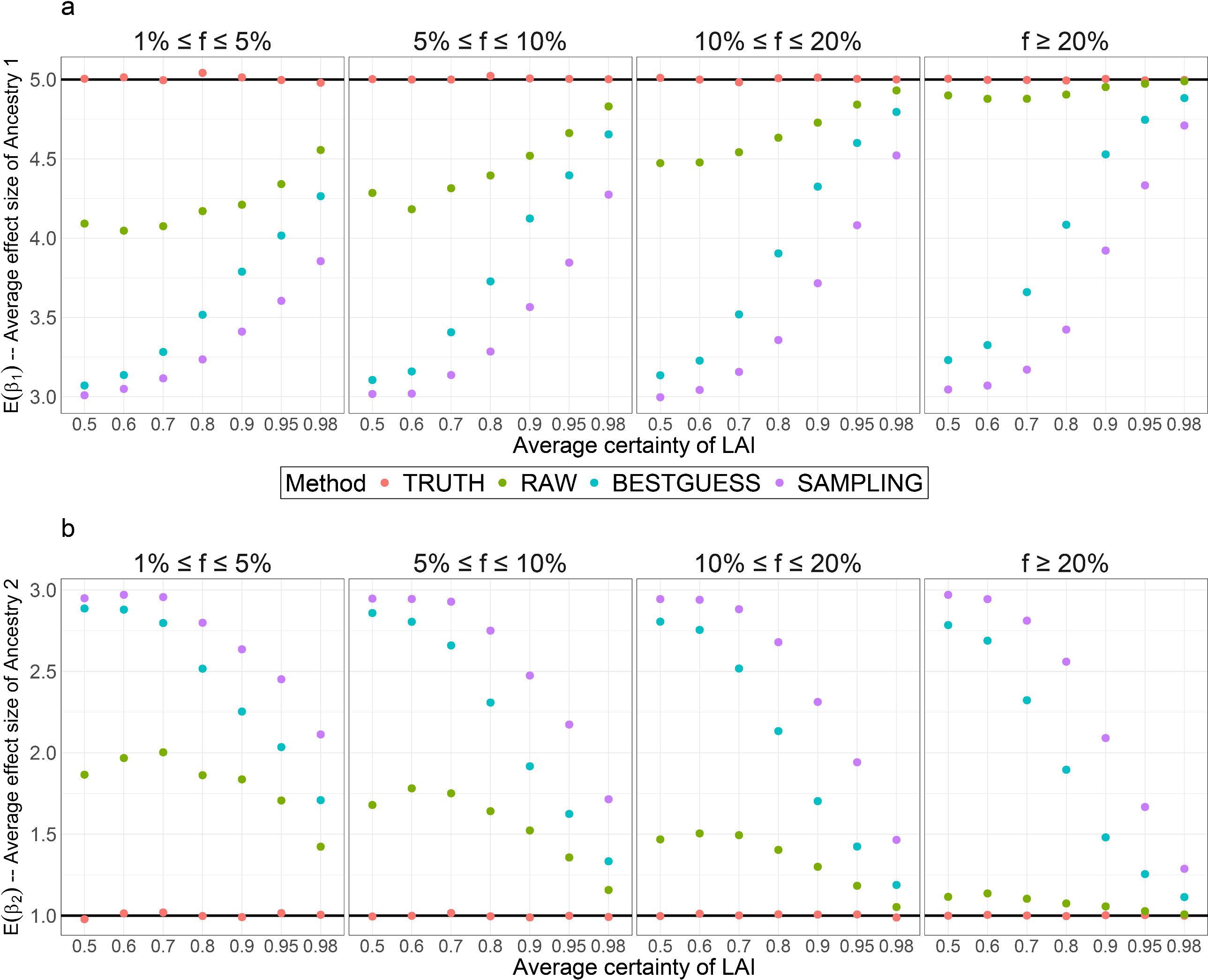

Supplement: S7 Fig — E(Ai1d*) denotes the average probability of Ancestry 1. The x-axis represents the average certainty of LAI (see Methods) and the y-axis represents the average estimated effect sizes for Ancestry 1 (plot a) and Ancestry 2 (plot b). Different MAF thresholds f are compared: 1%≤f≤5%, 5%≤f≤10%, 10%≤f≤20%, and f≥20%. The simulation was repeated 1,000 times with n = 20,000 diploid individuals. (TIFF) [file pgen.1011883.s011.tif]

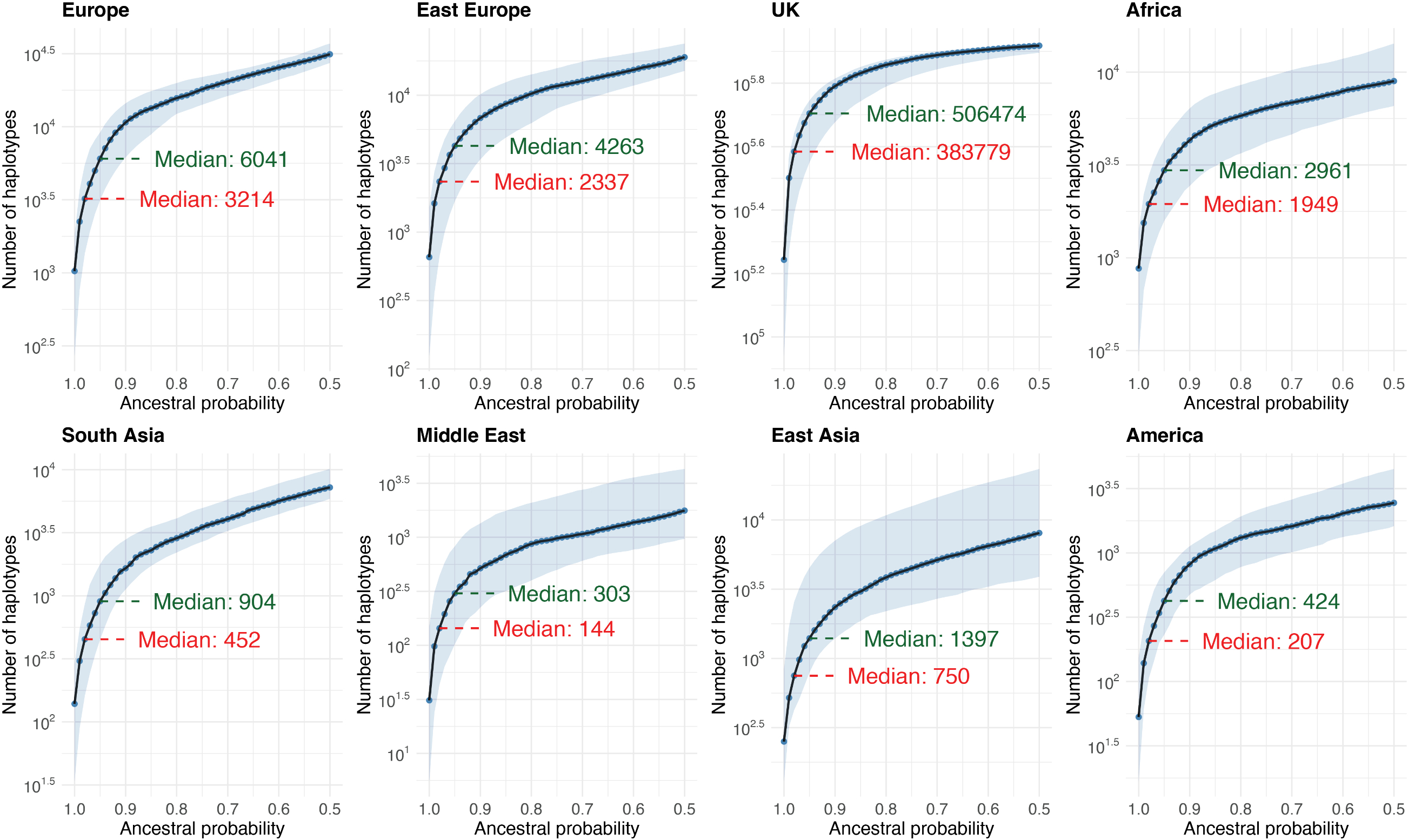

Supplement: S8 Fig — The x-axis represents the local ancestry probabilities, and the y-axis represents the number of haplotypes measured as two times the number of individuals painted in the UK Biobank on a log10 scale. There are a total of n = 925,388 haplotypes. Each plot displays the median cumulative haplotype count (blue dashed line) along with the corresponding 95% confidence intervals (shaded regions) at varying local ancestry probability thresholds, based on all pruned significant SNPs across 35 phenotypes (investigated in the ARS analysis) for 8 ancestries. Median values at the 98% (red dashed) and 95% (green dashed) probability thresholds are annotated. (TIFF) [file pgen.1011883.s012.tif]

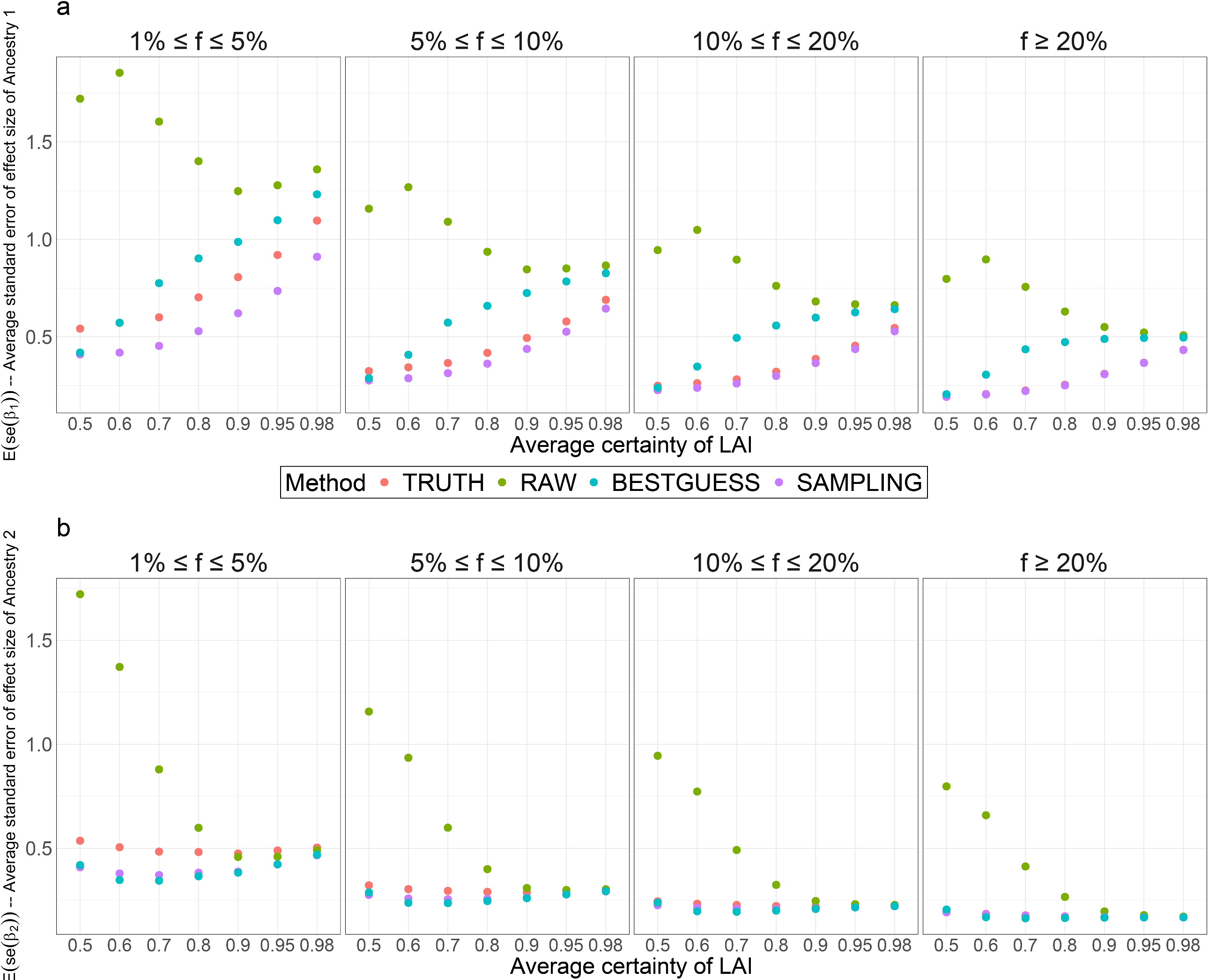

Supplement: S9 Fig — E(Ai1d*) denotes the average probability of Ancestry 1. The x-axis represents the average certainty of LAI (see Methods) and the y-axis represents the average standard error of the estimated effect size of Ancestry 1 (plot a) and Ancestry 2 (plot b). Different MAF thresholds f are compared: 1%≤f≤5%, 5%≤f≤10%, 10%≤f≤20%, and f≥20%. The simulation was repeated 1,000 times with n = 20,000 diploid individuals. (TIFF) [file pgen.1011883.s013.tif]

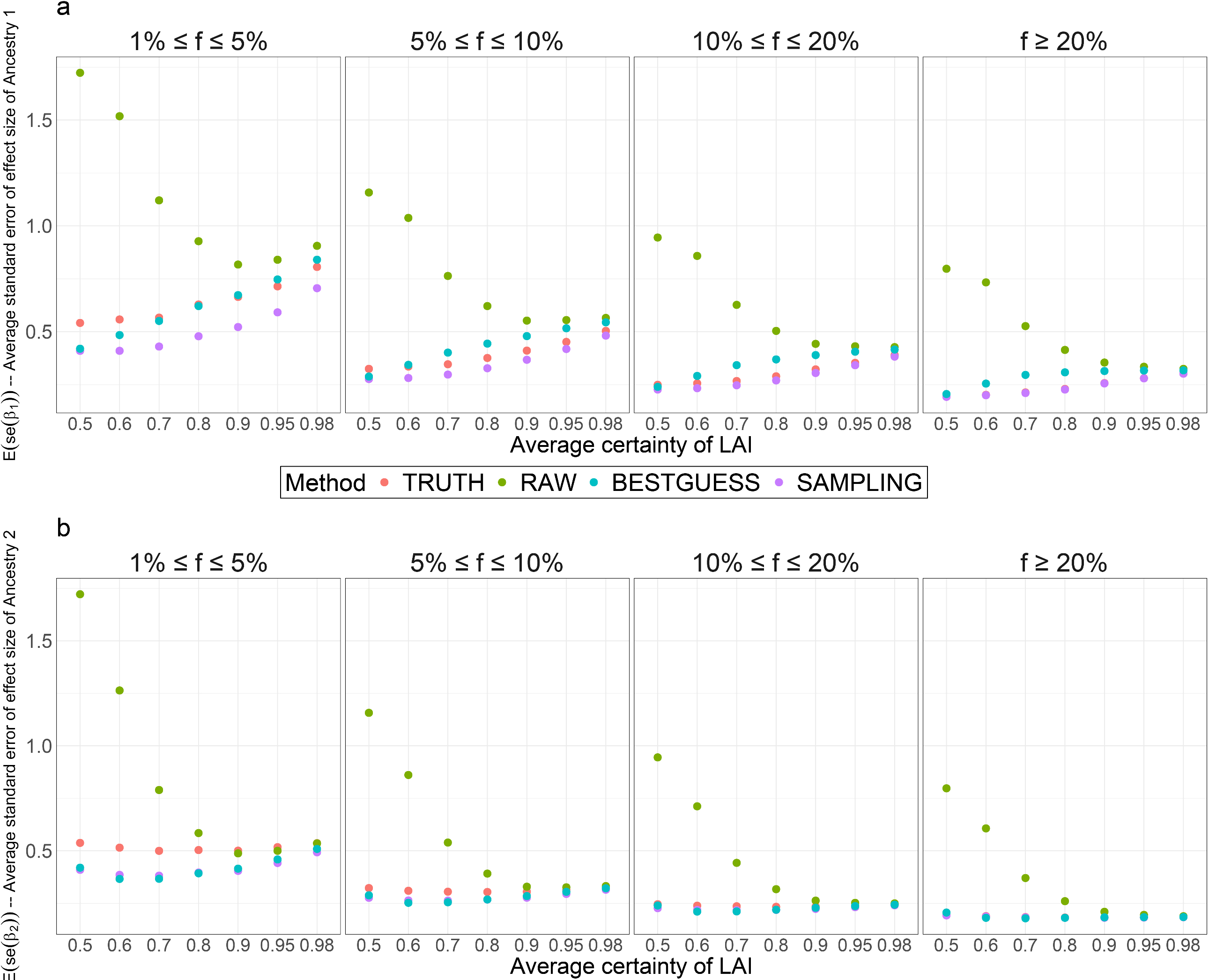

Supplement: S10 Fig — E(Ai1d*) denotes the average probability of Ancestry 1. The x-axis represents the average certainty of LAI (see Methods) and the y-axis represents the average standard error of the estimated effect size of Ancestry 1 (plot a) and Ancestry 2 (plot b). Different MAF thresholds f are compared: 1%≤f≤5%, 5%≤f≤10%, 10%≤f≤20%, and f≥20%. The simulation was repeated 1,000 times with n = 20,000 diploid individuals. (TIFF) [file pgen.1011883.s014.tif]

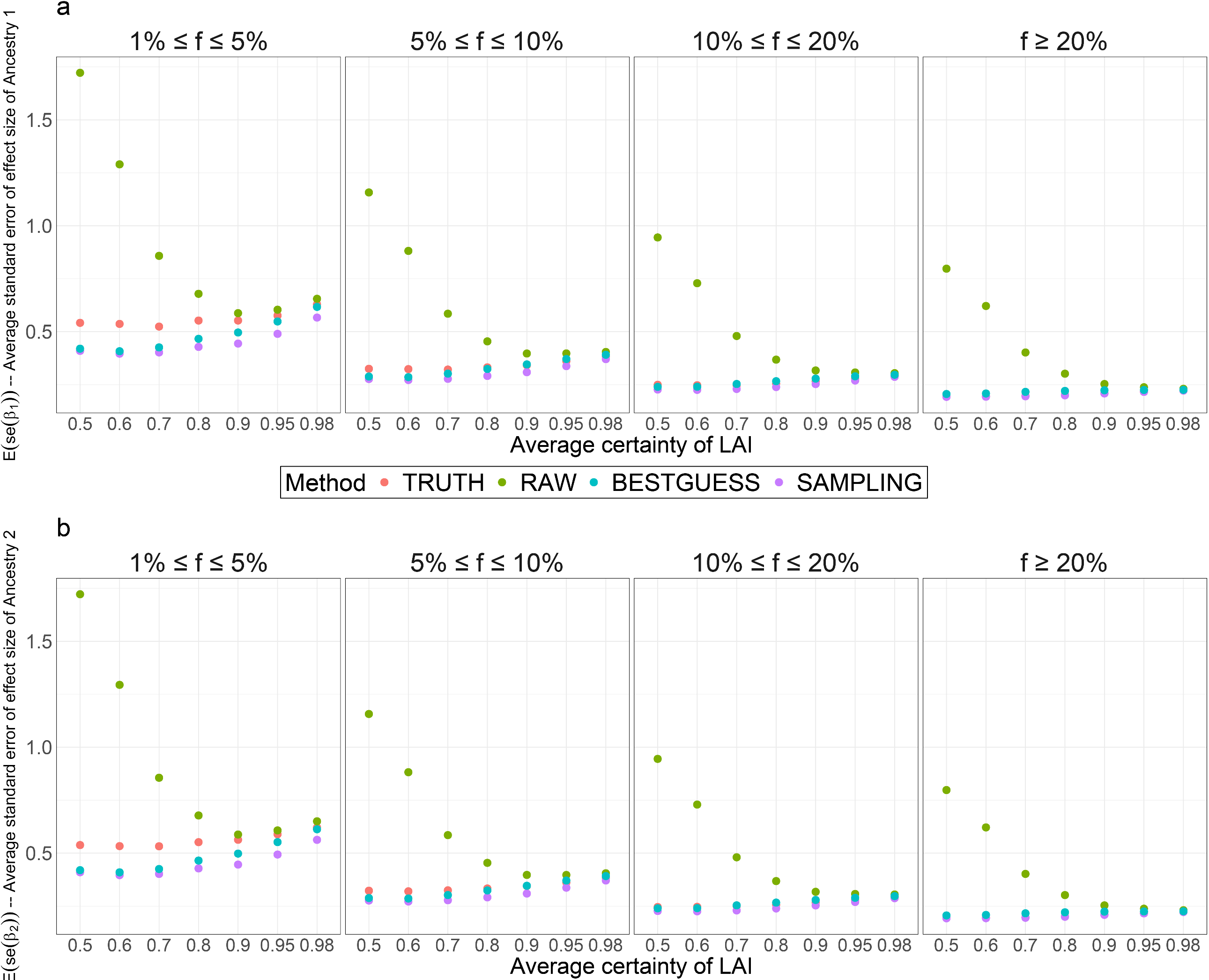

Supplement: S11 Fig — E(Ai1d*) denotes the average probability of Ancestry 1. The x-axis represents the average certainty of LAI (see Methods) and the y-axis represents the average standard error of the estimated effect size of Ancestry 1 (plot a) and Ancestry 2 (plot b). Different MAF thresholds f are compared: 1%≤f≤5%, 5%≤f≤10%, 10%≤f≤20%, and f≥20%. The simulation was repeated 1,000 times with n = 20,000 diploid individuals. (TIFF) [file pgen.1011883.s015.tif]

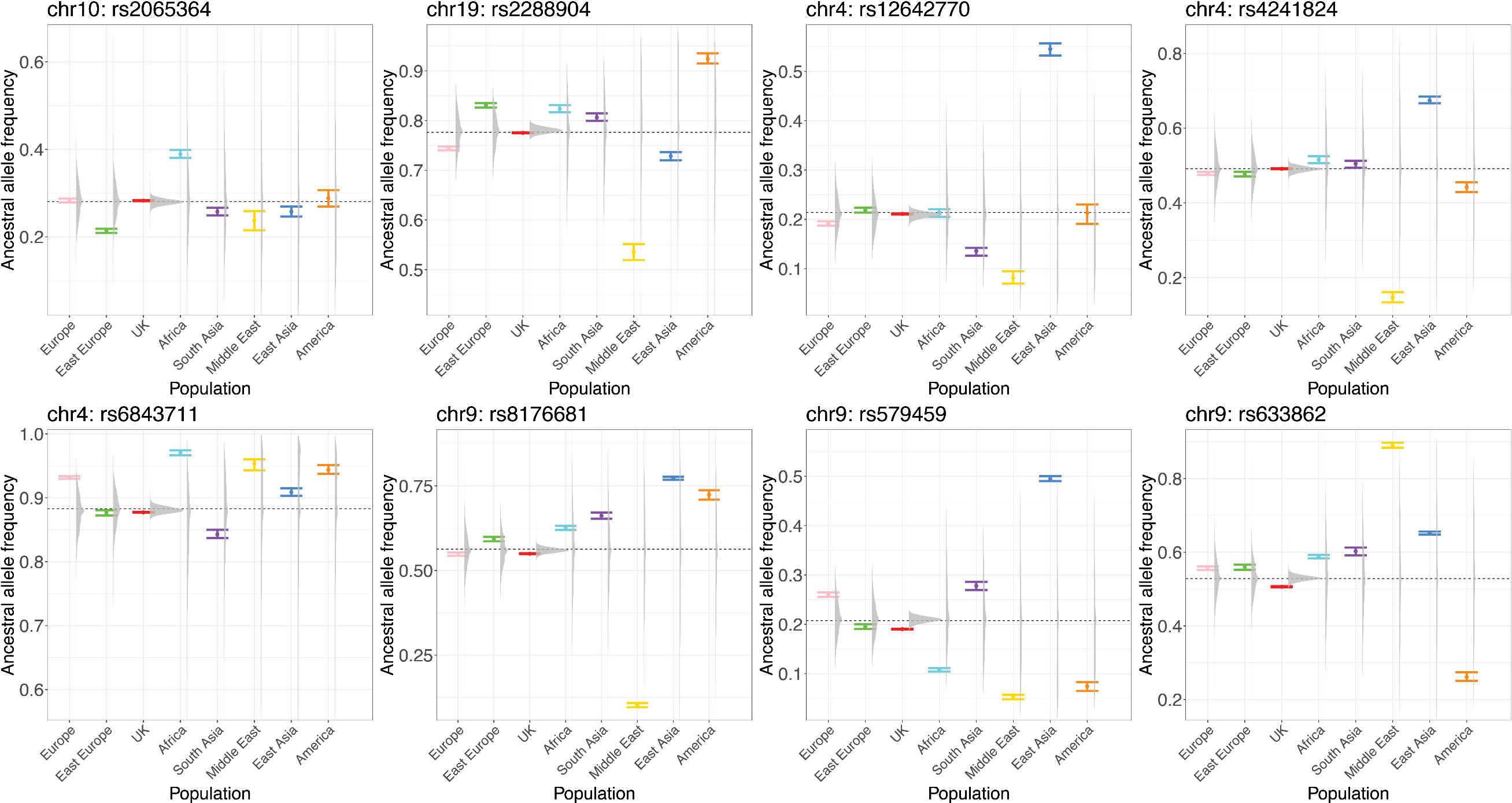

Supplement: S12 Fig — The error bars represent the 95% confidence interval of ancestral allele frequency. The distribution of simulated allele frequency for each population of all the matched SNPs in the UK Biobank is shown as a raincloud plot. The dashed black lines represent the genome-wide allele frequency. (TIFF) [file pgen.1011883.s016.tif]

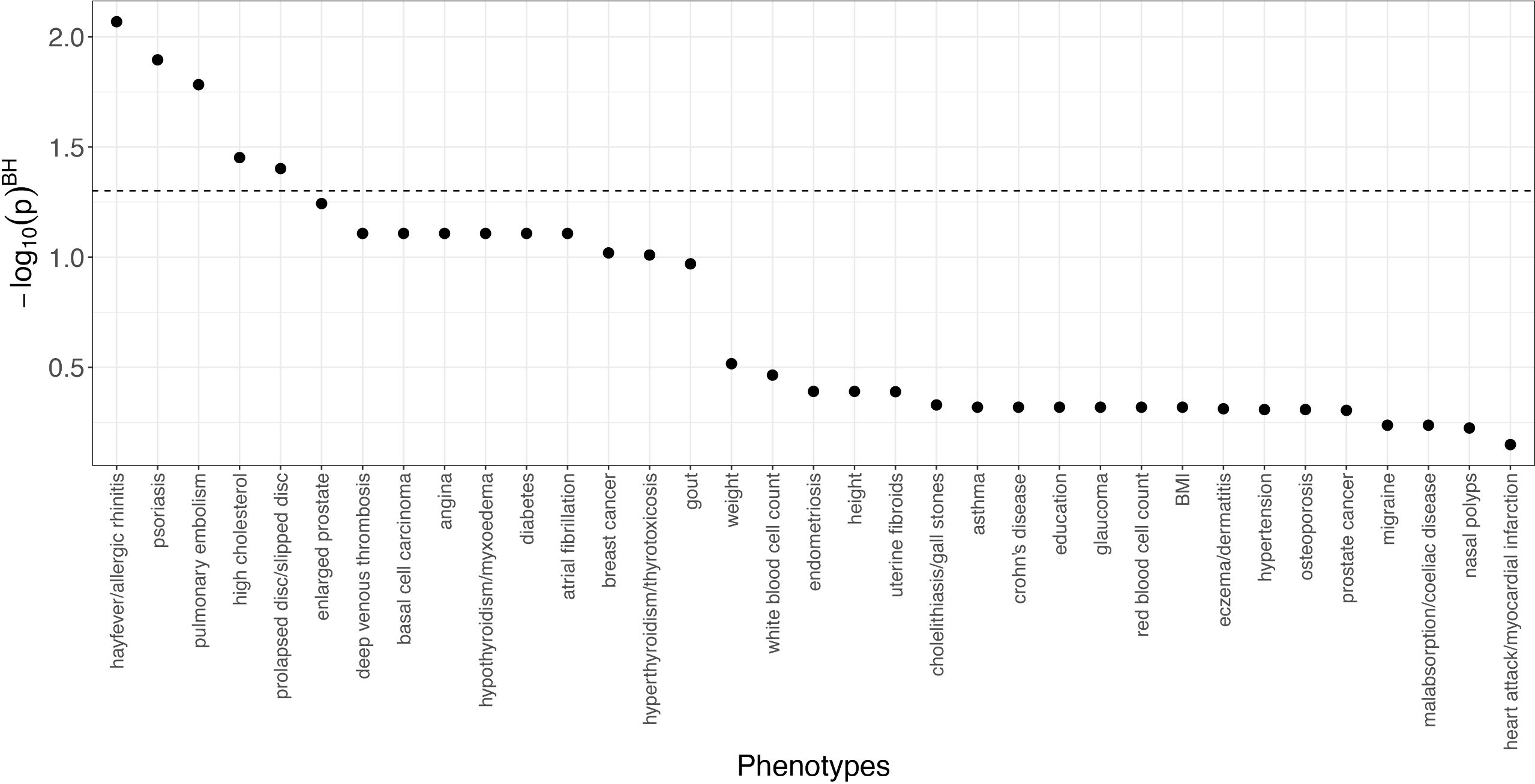

Supplement: S13 Fig — The y-axis shows the -log10 scale of the p-values derived from the ARS calculated across different populations and then aggregated using Fisher’s method, i.e. chi-squared test (see Methods). (TIFF) [file pgen.1011883.s017.tif]

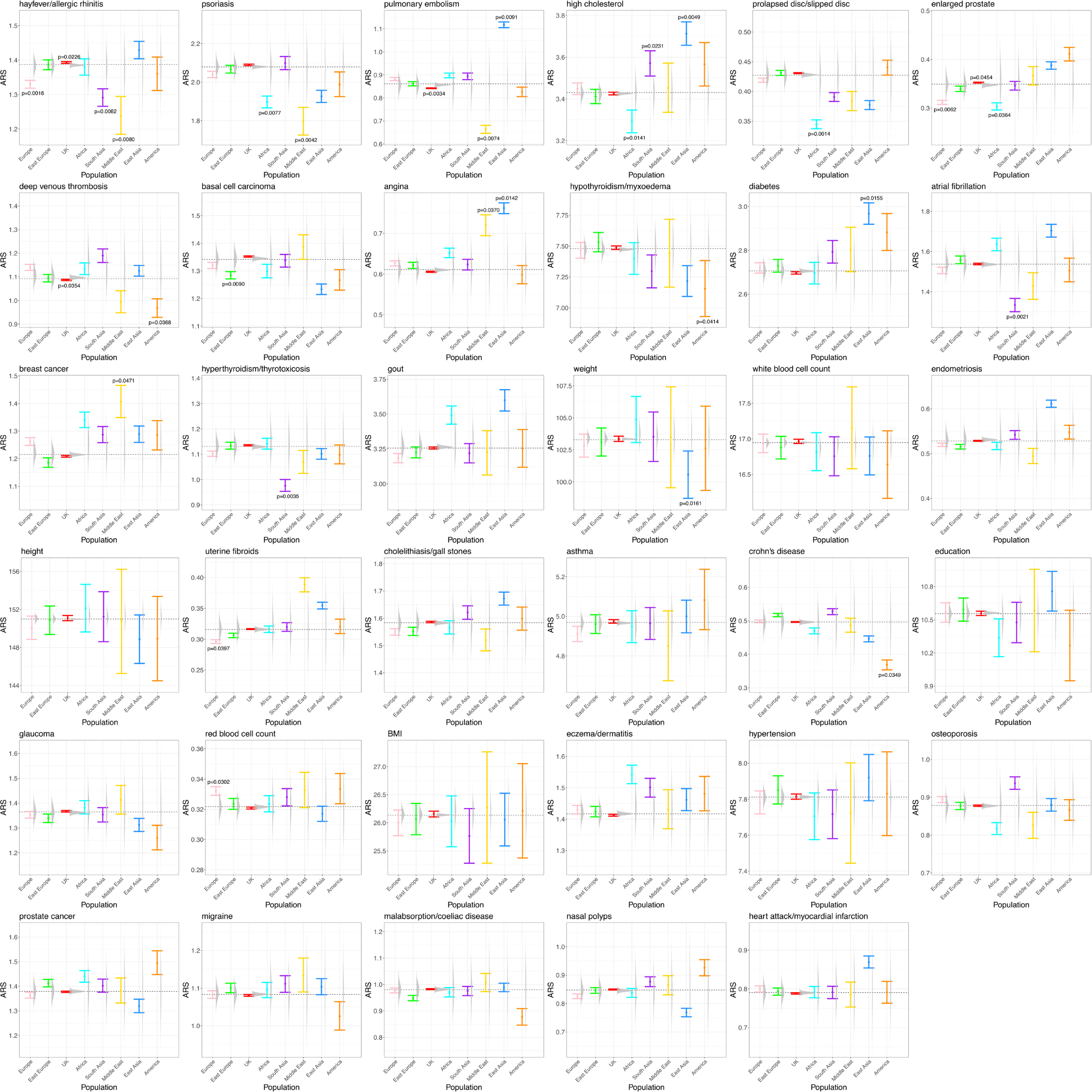

Supplement: S14 Fig — ARS are computed from n = 462,694 individuals in the UK Biobank. The error bars represent the 95% confidence interval of ARS. The distribution of simulated ARS for each population is shown as a raincloud plot. The dashed black lines represent the average ARS of all populations weighted by the population sizes. The ARS of populations with raw p-values significant at 5% computed from the two-sided empirical test are annotated. (TIFF) [file pgen.1011883.s018.tif]
