# Supplementary material for: From individuals to ancestries: Towards attributing trait variation to haplotypes
Source: PLoS Genet. 2025 Sep 30;21(9):e1011883. doi: 10.1371/journal.pgen.1011883 (PMC12507255; doi:10.1371/journal.pgen.1011883)
Supplement: Attachment — Submitted filename: response.pdf [file pgen.1011883.s019.pdf]

Reviewer's Responses to Questions

Our response is provided in blue, original comments in black. We have provided a “Revised Article with Changes Highlighted” at the end of the reviewer pack, which contains changes annotated in red.

Comments to the Authors:
Please note here if the review is uploaded as an attachment.

Reviewer #1: This manuscript explores the utility of haplotype-based representations, specifically haplotype components (HCs), for improving population structure inference and trait association studies in human genetics. Leveraging UK Biobank data, the authors compare HCs with traditional principal components (PCs) in a variety of contexts: birthplace prediction, phenotype prediction, GWAS correction, ancestry-specific effect size estimation (via Tractor), and the development of Ancestral Risk Scores (ARS). The authors argue that HCs provide more nuanced representations of population structure and that ARS, rather than ancestry-specific effect estimates, may be more robust in admixed populations. Overall, this manuscript is well-written. The proposed simulation studies and real data analyses support their conclusion. I have a few comments as follows.

Response: We thank the reviewer for their care and attention, as well as their supportive words. Detailed responses are inline.

1. The analyses focus almost exclusively on the UK Biobank, where the vast majority of participants are of British ancestry. The authors could discuss how the performance of HCs may vary across more admixed or globally diverse datasets (e.g., All of Us or PAGE). Additional simulations mimicking more complex admixture scenarios would strengthen this claim, while I understand it could be of future work.

Response: The reviewer has identified a big challenge, due to the way access to diverse data is usually (not) shared, and we agree with the criticism that we are underpowered with UK Biobank for admixture. We are trying to access more diverse data, which requires collaboration and we hope these results will give data owners a reason to engage with us.

We report these "minimal" simulation design to make the central point, which is that even in the simplest possible case the available methods simply don't work. This was enough to destroy our ambition to perform Tractor-like analysis for fine-scale UKB results and made us rethink the purpose of the project. We therefore canned the planned more complex simulations - those presented here build on experience from Ref [17] which included up to 100 populations and more complex admixture.

To address this for readers, we have inserted the text:

"To evaluate whether these results would be meaningful, we simulated local ancestry with uncertainty under the same linear regression model used for inference in a minimal two-way admixture model. Inference failure under this model means that causal effect sizes are not separately identifiable from local ancestry effects." (Line 187)

We also address the bigger picture in the discussion (Line 265):

"As datasets grow in scale to include a moderate fraction of a population, such as seen in the Estonian Biobank (200k of the 1.3M population), FinnGen (500K of 5.6M) or Our Future Health (working towards 5M of the UKs 69M) HCs promise to bring familial level prediction of population structure. Similarly, in more diverse and admixed populations such as PAGE, All of Us (currently 245k people from the USA) and the Million Veteran Program, methods that can identify local ancestry structure at scale are essential. This is true regardless of whether local ancestry is itself predictive, as explored by TRACTOR-like models, or just a proxy for confounding by LD as evidenced by models but without access to large diverse data."

2. While HCs outperform PCs in various predictive tasks, their biological interpretation remains unclear. For example, the authors mentioned that the optimal number of HCs is as large as 150 but for PCs is only 18. More explanation on interpreting the selected HCs would improve the generalizability of the results.

Response: That is a great suggestion, as we do have some limited biological interpretation to offer. We have added the following text:

"Unlike PCs which are distributed across all populations that share any historical genetic component (e.g. \cite{mcvean2009genealogical}), HCs \cite{yang2025sparse} form orthogonal patterns representing groups of individuals that share recent relatedness via genetic isolation or admixture. While this study focuses on the applications in the UK Biobank where the majority of participants are white British, applying the same methods in other biobanks with more diverse populations may show a greater advantage for HCs by separating recent relatedness in admixed groups from repeated admixture events." (Line 276)

3. The ARS approach is of potential interest, but the real-world utility is somewhat speculative, especially given that associated SNPs were primarily derived from European-centric GWAS. The authors should discuss more explicitly the implications of using ARS constructed from European-discovered variants when applied to other populations.

Response: This is a fair criticism and we try not to over-claim. We now discuss implications for ARS due to ascertainment as follows:

"Whilst ARS can be directly interpreted the same way that PGS are, they suffer from the same problems and potentially some new ones. Firstly, effect sizes are ascertained in Europeans and so predictive power decreases with genetic distance from this population, primarily due to LD\cite{hu2025fine}. Secondly, genome-wide distributions

of effects have shifted due to European drift from the historical African population\cite{ashrafGeneticDriftOutofAfrica2021}. Thirdly, unlike PGS, ARS work with inferred local ancestry which may interact with LD tagging of GWAS hits. The solution in all cases is to increase the diversity of GWAS panels, and to try to construct scores from fine-mapped, putatively causal effect size estimates." (Line 303)

4. The paper underscores the difficulty of accurate LAI and its impact on Tractor and related frameworks. It could be helpful for the authors to discuss how probabilistic LAI models (e.g. FLARE) can be leveraged for ancestry-specific effect size estimation.

Response: We agree - our results are agnostic on the model as long as it provides probabilistic assignments (some tools provide best-guess only, or by default). This is clarified in the text:

"Performance of estimates for effect sizes from LAI depend critically on uncertainty, leading to a need for LAI inference tools that estimate uncertainty, and leverage information - such as exploiting rare SNPs, and building models to address admixture in the panel - to reduce it." (Line 290)

Other comments:
1. Line 139, should it be "every PC > 18"?

Response: Corrected.

2. Line 334, "PC" should be "PCs"?

Response: Corrected.

3. Line 343, should it be "The top 150 HCs and 18 PCs"?

Response: For fairness, we only reported the results of GWAS correction with the top 18 HCs and PCs in the paper. We have also explored using 40 HCs/PCs, but we did not report the results because they look similar to 18 HCs/PCs correction, but contain some over-correction which makes interpretation less straightforward. The text was clarified.

Reviewer #2: Overall, I thought this was a timely, important and interesting paper, building on recent results published this year on the importance of haplotype-based methods for GWAS. The authors firstly confirm previous results that haplotype components are better at capturing population stratification than normal PCA analyses, and show that HCs have greater predictive power for a variety of traits because of this. However, they do show that despite this GWAS results for UKBB do not change much, which they hypothesise is because of the low amounts of structure. The completely novel results of the paper are in the second section on local ancestry inference and association studies, where through simulations the authors show that ancestry-specific GWAS is poor when LAI confidence is low. They present and discuss the use of Ancestral Risk Scores as an alternative for understanding population-specific risk.

The paper provides multiple novel results and statistics, building on methods presented in Yang et al., 2025. It opens up avenues of research using datasets with more complex ancestry patterns than UKBB, and I think will be of interest to the GWAS/ population genetics community. I have some minor comments to improve the clarity of the manuscript.

Response: We thank the reviewer for their supportive perspective and thorough reading. Our responses are given inline.

Abstract- I thought the only new GWAS hits were for education attainment- 'birthplace and lifestyle related phenotypes' seem misleading?

Response: A good issue to raise, as we didn't discuss the GWAS results very fully, not wanting to over-emphasise a "negative" result. On birthplace we did say: "Because HCs are highly informative of birthplaces, we found changes in significance when correcting for HCs/PCs in both the east and north coordinates in a number of SNPs." and the other comment was made based on Supp Fig 4-5, which we now explicitly discuss:

"The p-value distribution was shifted in other phenotypes, including Age started smoking in former smokers", "Tea intake", "Breastfed as a baby", "Alcohol usually taken with meals", "Cervical cancer" and "Cin/pre-cancer cells cervix". Whilst interesting, we don't view these as substantial differences in the UK Biobank dataset." (Line 179)

Line 47- check this ref, I think they are saying 40% of variation in traits is dues to SNPs, not 40% of associations are between SNPs and traits.

Response: Thank you for the correction. We have rewritten to:

"Whilst SNPs can predict trait variation - for example, 40\% of observed phenotypic variance is predictable by common SNPs and up to 90\% including 5M rare SNPs\cite{harris2024genome}, this may not be causal and taking a SNP-centric perspective on confounding population structure has limitations." (Line 47)

Fig 1- could you include some discussion on the birthplaces with very low predictive power- is it a sample size thing, are they always classified as a nearby country? Similarly there are a few cases where PCs seems to do better, e.g. Bulgaria and Croatia- any ideas on this, maybe they are cases with lots of recent migration between countries throwing off HCs? Overall caption of Fig 1 could use more detail on the analysis- 1d looks a lot like a PCA without reading all the text.

Response: Thanks for the prompt to explore this further! To address it we've added the following comment:

"The two countries for which PC predictions improve on HCs are Croatia and Palestine with 41 and 21 individuals respectively of the 20,092 total used (Supplementary Table

4), so this may reflect statistical noise or imperfect labels, as many individuals will trace ancestry back to places other than their location of birth." (Line 125)

It is possible that certain population structures such as being formed from a characteristic admixture proportion do exploit the "global" nature of PCs better than HCs when sample size is low. (as sample size grows, we'll hit upon a relatively recent relative and do much better with HCs.)

Examining the confusion matrix (Supplementary Figure 1) we see that HCs cluster Croatia with Serbia/Montenegro more than PCs (which oddly assign some probability to Cyprus and India!), and Palestine with Israel more than PCs. On reflection we've omitted further speculation from the paper, both due to political sensitivities and because the ranking is specific to the choice of metric (i.e. we score only correct country or not, rather than giving partial credit to getting the country close), but it does warrant further investigation.

We've corrected figure 1 labeling to explain it better:

"{\bf Predicting birthplaces using 150 HCs or PCs in a left-out sample of UK Biobank using xgboost.} a-c, prediction of birth Country for 20,092 internationally born non-British ancestry individuals, broken down by birth country. a-b, example countries, visualized in a world map. c, the recall rate (higher is better) contrasting HCs and PCs. d, visualising information about birth location present in HCs (left) or PCs (middle) using xgboost prediction of the UK birthplace of n=341,881 British-born individuals with self-reported British ethnicity, coloured by ancestral region of birthplace (right)."

Line 164- state how many HCs and PCs did you use for correction here- this information is only in supplementary. Also did you try the GWAS with >18 HCs, and see any changes in result?

Response: Changed to "To compare the effectiveness of HCs and PCs in correcting for population structure in GWAS, we performed GWAS using the top 18 components on 26 continuous and 53 binary phenotypes" (Line 168)

For the second question, we've also tried with 40 HCs/PCs correction. For 40 HCs correction, we did not see any changes in signal-detection; for 40 PCs correction, we find overcorrection as in ref. [16]. Therefore we did not report the result of 40 HCs/PCs correction.

Line 170- examples of other phenotypes?

Response: Assuming you mean the phenotypes associated with the SNPs we identify - there are many but some are too technical for us as statistical geneticists to comfortably interpret (e.g. https://www.genecards.org/cgi-bin/carddisp.pl?gene=CCNT2-AS1). We instead give more detail and reference for what makes intuitive sense (i.e. neurological pathways, Parkinsons). Interestingly, a recent non-peer reviewed meta analysis

https://www.medrxiv.org/content/10.1101/2025.07.02.25330781v2 pushes back against the well-established educational attainment/parkinsons link - we don't want to get into that discussion here as it feels like a distraction. Our updated text is:

"For educational attainment, we found 6 SNPs overlapping gene ACMSD associated with tryptophan metabolism and neurlogical disorders including parkinsonism \cite{marti-massoACMSDGeneInvolved2013})
and CCNT2-AS1 (associated with various disorders, again including Parkinson's disease \cite{changMetaanalysisGenomewideAssociation2017}) which were significant at genome-wide significance level with HC-corrected GWAS, but had no significance ($p>0.01$) with PC-corrected GWAS." (yes the literature intentionally separates "parkinsonism" from "Parkinson's disease"... Line 171)

Line 180- this section could use just a few more sentences describing what you did to improve clarity- a little more about your simulations, and an explanation of truth, raw etc would be useful (and why you picked these).

Response: To try to make things more explicit we now say:

"we simulated local ancestry with uncertainty under the same linear regression model used for inference in a minimal two-way admixture model". (Line 188)

and added explanations of the representations:

"TRUTH uses the real LAI, RAW uses the certainty on LAI calls as a weighting, BESTGUESS treats the highest probability LAI call as a certain call, and SAMPLING randomly averages the effect size estimate from many random samples from the probability distribution (see Methods)." (Line 193)

Additionally the results from supplementary fig. 8 seem very important for explaining the context of your simulation result but are poorly explained- I think you used sparsepainter for the probabilities but this isn't explicitly mentioned anywhere? The total number of haplotypes that exceed >0.5 probability is different for each continent depending on the sample, so could you report this alongside the median? Maybe some (very brief) background on different LAI methods and their accuracies could be useful here. Overall I'm still a bit confused- it seems TRACTOR approaches do have merit in datasets of admixed inds with ancestry from multiple continents (where LAI should be relatively accurate), but UKBB does not have many of these individuals (or of non-UK individuals in general) AND LAI probs are lower, so will have low power for ancestry specific effects?

Response: We agree that TRACTOR can work when calls are confident - "The Tractor framework can always return accurate estimates of effect sizes on average (Fig. 4196 and Supplementary Figs. 6-7) when we know the true ancestries (TRUTH)" (Line 196). It will likely be possible for Africa/European/Asian (and Papuan) if sufficient datasets are available, but at no finer resolution (in particular, we lack good reference data for the

Americas as 1000G American individuals contain European admixture). We now say (Line 282):

"Where uncertainty can be eliminated - perhaps at the intercontinental level through careful curation of reference data and stringent thresholds - LAI-specific estimates can in principle be obtained, though very large sample sizes will be needed \ref{mesterImpactCrossancestryGenetic2023a}."

We also properly explain the details of the LAI results on UK Biobank:

"\hl{This is not just a theoretical concern. Estimating LAI for the UK Biobank dataset with SparsePainter (which has comparable accuracy to other LAI estimators \cite{yang2025sparse}), and using 1000 Genomes as reference}, few haplotypes outside of Europe meet the threshold of 95\% and 98\% LAI probability (Methods, Supplementary Fig. \ref{fig:hap_distri}). \hl{Specifically, we examined the LAI from all 925k haplotypes and averaged across all pruned significant SNPs from the 35 phenotypes investigated in the ARS analysis. Retaining "certain" calls at 98\% certainty we obtain a sample size of just 144 Middle East, 207 America, 452 South Asian, 750 East Asian, and 1949 African LAI segments (rising to 303, 424, 904, 1397, and 2961 respectively at 95\% confidence). Uncertainty is costing a lot here - the number at only 50\% confidence rises to 1.8K, 2.5K, 6.3K, 7.1K and 8.9K, respectively. The number of confidently called LAI segments is far too small for any GWAS,} indicating the limited power of the UK Biobank to implement the Tractor framework in practice effectively." (Line 202)

Line 211- Again, just another sentence or two on ARS and how they are calculated would be useful at the start of this section for context. The A_ijk used in the ARS calculation are the same as those shown in Fig s8?

Response: Yes, that is correct. We have clarified (Line 229):

"To work with quantities that are uncertainty-tolerant, we painted the UK Biobank with a public-available reference panel with 93 populations that we summarized (Supplementary Table 3), and then estimated the frequency of each SNP in each ancestry (Methods) which results in an ARS when included as weights in a sum over effect sizes for a phenotype."

Did you try ARS on your smaller reference groupings, rather than just the continental ones?

Response: We did calculate ARS on fine-scale population estimates. Whilst there were some interesting results, the very low sample sizes led to many cases where the most extreme (and visually obvious) results were not interpretable, nor significant after bootstrap resampling. We concluded that with the power available, the differences in fine-scale results didn't reflect biologically meaningful variation and that larger sample sizes from diverse populations would be necessary to make e.g. visualisations of clines of ARS across Europe.

Line 299- was test and training set randomised at each repeat?

Response: Yes - we clarify "This entire CV process was repeated 50 times" (now Line 343).
